# Seeking community water fluoridation information on state health department websites

**DOI:** 10.1371/journal.pone.0251139

**Published:** 2021-05-20

**Authors:** Catherine Maybury, Matt Jacob, Jessica M. Flanders, Alice M. Horowitz

**Affiliations:** 1 Center for Health Literacy, University of Maryland School of Public Health, College Park, MD, United States of America; 2 Jacob Strategies LLC, Washington, DC, United States of America; 3 Department of Behavioral and Community Health, University of Maryland School of Public Health, College Park, MD, United States of America; University of Queensland, AUSTRALIA

## Abstract

Community water fluoridation (CWF) is the most effective and equitable approach to preventing dental caries (tooth decay). Yet millions of Americans, especially those at highest risk of caries, do not know what CWF is or its preventive benefits. State health departments are responsible for educating their respective populations. Thus, this study assessed health department websites (N = 50) to determine if CWF content existed, the ease of finding it, and if it was written in plain language and for a consumer audience. We used the web component of the HLE2: The Health Literacy Environment of Hospitals and Health Centers (HLE2) to assess how easy or difficult it was to the navigate a website and find information. Forty-one websites had CWF information; 37 states had content written for a consumer audience. HLE2 scores ranged from 0 to 54 points (60 possible). Only five states had websites with a HLE2 score of 50 or higher. SHDs with higher HLE2 scores were easy to navigate and their content was written for a consumer audience. Study findings suggest most SHDs should improve their website’s CWF content and its accessibility to better promote the role of fluoridated water in preventing dental caries.

## Introduction

Over 75 years of scientific research has proven that optimally fluoridated water is the most cost-effective, safe and equitable way to prevent dental caries [1–6]. Community water fluoridation (CWF) helps reduce health disparities [7]. When communities choose to fluoridate their public water supply, everyone who consumes the water benefits regardless of age, income or level of education [8]. The U.S. Centers for Disease Control and Prevention (CDC) named CWF one of 10 great public health achievements of the 20th century, and the agency continues to recommend this public health practice [9]. Importantly, recent studies show that dental caries (tooth decay) and the associated treatment costs increased significantly after communities discontinued water fluoridation [10–12].

Seventy-three percent of the U.S. population on public water systems, approximately 207 million people, have access to fluoridated water [13]. Yet dental caries remains one of the most common chronic diseases with more than 20% of Americans having untreated tooth decay [14, 15]. Despite its prevalence, dental caries can be prevented through the appropriate use of fluorides, application of dental sealants, healthy dietary habits, good oral hygiene that includes regular tooth brushing with fluoride toothpaste and seeing a dentist regularly [16, 17]. Unfortunately, many individuals, especially those with lower levels of oral health literacy (OHL), do not know how to prevent dental caries [18, 19].

We consider OHL to include the knowledge, understanding, and practices to promote oral health and engage in behaviors that prevent dental diseases such as dental caries [20]. Many individuals with low OHL do not know what fluoride is, what it does or why it is important to drink fluoridated water. Drinking fluoridated water reduces dental caries by approximately 25% in children and adults [21]. A 2018 study concluded that “oral hygiene in the absence of fluorides has failed to show a benefit in terms of reducing the incidence of dental caries [22].” Nonetheless, Gift et al found that 70% of Americans claimed the best way to prevent dental caries is brushing and flossing, and only 7% of respondents correctly identified fluoride [23]. Horowitz et al. state that 98% of adults were aware of fluoride but only 58% were aware of its purpose [18]. From a public health perspective, we are concerned about individuals with low OHL because they have poorer oral health outcomes than those with higher OHL [24–27].

Raising OHL is especially important because the public often encounters false or misleading information about CWF online or through social media [28]. Canadian physicians decried the “torrents of misinformation” that circulate about fluoridation, vaccinations and other health and science topics [29]. The incidence and prevalence of dental caries strongly suggests the public needs to better understand that CWF is a crucial form of prevention. Concomitantly, the public needs credible sources for information about CWF.

State health departments have a mission to advance community health by providing health services to their constituents, and one key service is accurate and timely health information that promotes disease prevention [30]. Indeed, as the nation rapidly shifts to a consumer-driven communications environment, experts urge health departments to embrace “the public health role as interpreter and distributor of information [31].” With eight in 10 adult internet users reporting they search online for health information [32, 33], Google and social media platforms have sought to combat COVID-19 misinformation by directing people to the websites of the CDC and other trusted public health organizations [34]; this strategy has the potential to encourage more Americans to view health department websites as an appropriate source for other topics that are often the subject of deceptive or confusing online messages. For all of these reasons, CWF is one topic that should be on all state health websites.

Additionally, the way in which health department websites write and organize their content can make it more challenging for people with low OHL to find information. As federal health officials have observed, people with limited literacy skills can struggle to navigate a website [35–39]. The health educators and navigators who advocate for the most vulnerable people also need sources for accurate health information that are user-friendly. In an Oregon study, public health nurses viewed the state health department’s website as a trusted source but struggled to find desired information on the site [40]. As health communication experts have noted, improving the quality of health-related websites “has the potential to improve the health literacy—and the health—of the population [41].” For all of these reasons, state health departments should prioritize the accuracy and accessibility of content.

The purpose of this study was to assess all 50 state health department (SHD) websites for content about CWF and its role in preventing dental caries and the ease or difficulty of finding this information.

## Materials and methods

This cross-sectional study used the web component of Rudd and colleagues, HLE2: The Health Literacy Environment of Hospitals and Health Centers (HLE2), to evaluate SHDs’ oral/dental web pages for CWF content [42]. We did not include the District of Columbia in our evaluation. The HLE2 web component indicates how easy or difficult it is to navigate a website and find information. It focuses on general features of the home page such as having a simple search function and links to major sections of the site; and organizational qualities such as a main message statement and meaningful groupings for content.

The HLE2 18-item web assessment uses a 5 point Likert-type scale with values of 0 (Never–not practiced yet), 1 (Rarely–practiced less than 25% of the time), 2 (Occasionally–practiced less than 50% of the time), 3 (Frequently–practiced about 75% of the time), and 4 (Always–practiced almost 100% of the time). Scores can range from 0 to 72, but three items that assess mathematical calculations did not apply to the CWF content and they were removed from the assessment per the HLE2 author’s directions. Thus, for this assessment scores could range from 0–60. For the evaluation, one team member assessed five states and then reviewed the results with another team member to calibrate the assessment. The differences between the two raters were few and minor.

The team members then assessed the remaining websites separately and discussed results to resolve differences. We categorized the HLE2 scores as high (80th percentile or higher), medium (70^th^ to 79^th^ percentile) or low (less than 70^th^ percentile) for reporting purposes.

**Fig 1:**
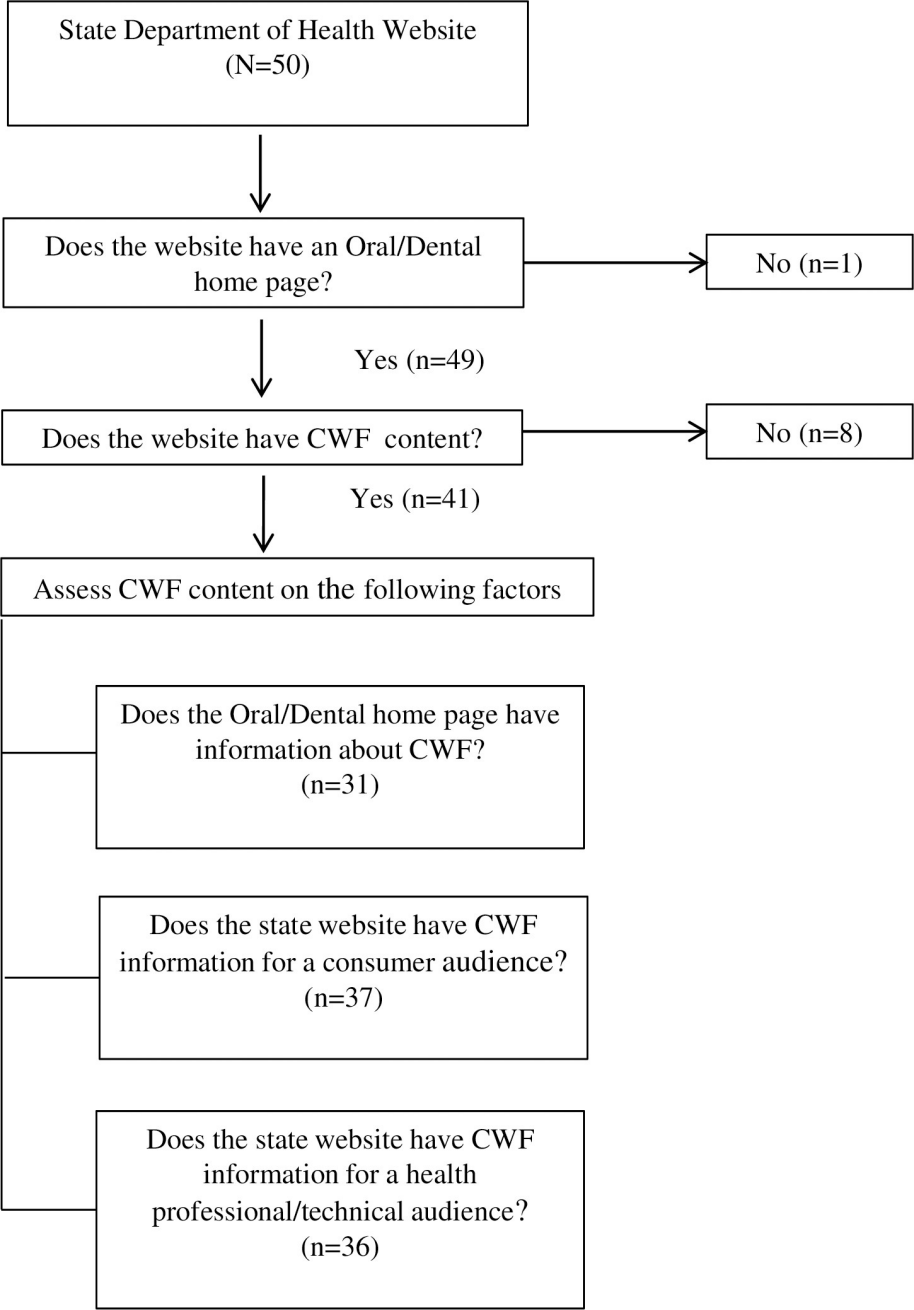
Process for evaluating U.S. state health department websites (N = 50) for content about community water fluoridation, August 2019.

Our assessment’s second phase examined CWF content starting with a Google search to find each SDH website. Next, we used the site’s navigation to reach the office of oral/dental health (ODH) homepage and determine if the page contained CWF information. Water fluoridation is foundational to preventing dental caries, so we would expect it to be highlighted on the ODH home page. If the ODH homepage lacked CWF information, we assessed how many clicks it took to reach this content. If CWF content is not on the ODH homepage or is several clicks away, users may not find the information [43]. We noted if a state’s website had no content about CWF or if the website only had links to external resources. Our search terms were CWF, fluoridation, fluoridated water, and fluoride. For states with CWF content, we determined if the information was written for a consumer or health professional/technical audience. Content was categorized as being for a consumer audience if the website specified the content was for consumers and if the content was written using plain language principles [44]. Plain language is writing that is clear and concise and allows the audience to “find what they need, understand what they find, and use what they find to meet their needs [44].” Plain language design principles include organizing the content into sections with meaningful headings, using lists to break up the text, using images and writing in short sentences [45]. Content was categorized as being for a health professional/technical audience if it had terms and information suited for health care providers and/or water operators. We also noted if SHDs linked to external websites such as CDC, American Dental Association or American Academy of Pediatrics (Fig 1).

## Results

Forty-one states had CWF information on their department of health website; nine lacked any CWF content. Thirty-one states had a link for CWF on the ODH homepage. Thirty-seven states had consumer-focused content while 36 states had content for a health professionals/technical audience. Ten states without health professional/technical content on their website linked to a CDC webpage. Almost half (n = 24) of the states had links for both audiences. It required between two and seven clicks to reach the ODH homepage from each state’s SHD homepage. Most (n = 27) required two clicks, and these websites were easy to navigate. Two states that were difficult to navigate required five and seven clicks respectively to reach the ODH homepage (Table 1).

**Table 1:** U.S. state health department website (N = 50) navigation characteristics relating to community water fluoridation content, August 2019.

Website Characteristics	n (%)
The state health department website has information about CWF	
Yes	41 (82.0%)
No	9 (18.0%)
The Oral/Dental home page mentions CWF	
Yes	31 (62.0%)
No	19 (38.0%)
The number of clicks from the health department home page to the Oral/Dental homepage (range 2–7)	
2 clicks	27 (54.0%)
3–4 clicks	21 (42.0%)
5 or more clicks	2 (4.0%)
The number of clicks from the Oral/Dental home page to CWF information	
0–1 clicks	32 (64.0%)
2 or more clicks	9 (18.0%)
No CWF page	9 (18.0%)
Type of audience CWF information is written for	
A consumer audience	37 (74.0%)
A health professional/technical audience	36 (70.0%)
Website has links to external evidence-based information about CWF	
Links for both consumer and health professional/technical audiences	24 (48.0%)
Links for a health professional/technical audience only	10 (20.0%)
Links for a consumer audience only	2 (4.0%)
No links to external websites	14 (28.0%)

**Fig 2:**
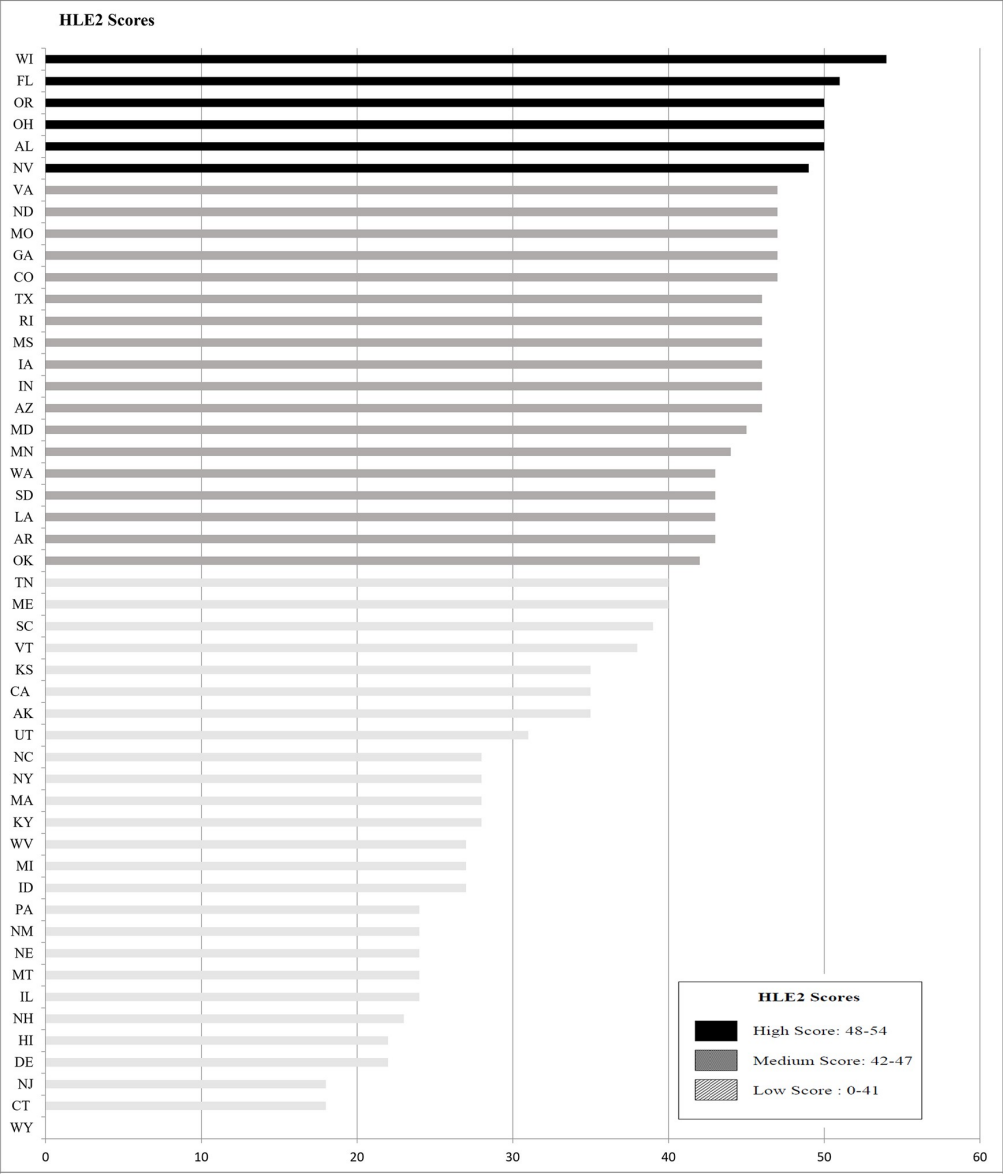
State health department (N = 50) HLE2^a^ scores assessing website content related to community water fluoridation, August 2019. ^a^ The HLE2 Score is an indicator of how easy or difficult it is to navigate a website and find information.

For the second part of our study, the HLE2 web assessment scores ranged from 0 to 54 out of a possible 60 points (Fig 2). Six states had high HLE2 scores, but only one state was in the 90^th^ percentile. The six states (Wisconsin, Florida, Ohio, Oregon, Alabama and Nevada) were fairly diverse in terms of their region and their ranks in the percentage of residents who have access to CWF. Overall, states with the highest HLE2 scores had websites that were easy to navigate and the CWF content was written for a consumer audience.

Eighteen states had medium HLE2 scores, and 26 states had low scores. Low-scoring states generally had websites that were more difficult to navigate (Arkansas, Connecticut, Hawaii, Maryland, Massachusetts and North Carolina) and offered little or no CWF content. States with the lowest HLE2 scores did not have a CWF page (Hawaii, Idaho, Illinois, Montana, Nebraska, New Hampshire, New Jersey, Pennsylvania, West Virginia and Wyoming). Some low-scoring states with no CWF information on their website provided links to CDC fluoride-related web pages [9].

## Discussion

For the public to understand CWF information, the website content must be scientifically accurate and written in plain language. Plain language is critical because the National Assessment of Adult Literacy (NAAL) found only 12% of adults have proficient health literacy [46], meaning only a small percentage of U.S. adults understand health information well enough to take steps to promote health and prevent diseases such as dental caries. Further, websites should be designed so users can easily find the information they are looking for. A majority of states (n = 41) had CWF content on their SHD websites. However, only six states had HLE2 scores in the 80^th^ percentile or greater for navigability and CWF content. The remaining 44 states’ HLE2 scores indicated SHD websites with insufficient information about CWF for a consumer audience and/or poor navigability.

States with higher HLE2 scores were easy to search and navigate. For example, Colorado’s CWF content was written in plain language, had links to consumer-oriented websites such as the Campaign for Dental Health, managed by the American Academy of Pediatrics [3] and had information about CWF for both consumer and health care/technical audiences [47]. Georgia also had CWF content written in plain language, including content on how fluoride works and its benefits, as well as links for both consumer and health care/technical audiences [48]. These two state websites serve as models for how to present CWF information.

Nine states, including Wyoming and New Jersey, had no CWF information on their websites, representing a missed opportunity to provide information that empowers consumers to manage their health. It is possible that health officials in a few SHDs (Hawaii and New Jersey) could have made deliberate decisions not to include CWF on their web pages because only a small portion of their populations have access to fluoridated water and because they do not expect these portions to increase [13]. Yet we would contend that a lack of such content and/or poor accessible to such content misses an opportunity to educate the public about evidence-based health practices, rendering it more difficult for health advocates to secure policy change that might expand the prevalence of local fluoridation programs.

Other states such as New York and Montana only had links to CDC’s website and they did not provide a description of CWF or the content that was being linked to. Providing links to websites with no explanation of its content, especially if the link is to technical information, may make the content less inviting. Interestingly, Montana’s ODH homepage encouraged residents to eat a healthy diet “and reduce the amount and frequency of sugary beverages”—language that could have been easily amended to include the benefits of drinking tap water that is fluoridated [49].

Many states including Massachusetts and Maryland had information about CWF, but the websites were hard to search and navigate, requiring 4–7 clicks to reach the CWF content. These states could benefit by following expert guidelines on web design by federal agencies, which included this insight: “The more decisions that users are required to make concerning links, the more opportunities they have to make a wrong decision [50, 51].” These guidelines also mention the importance of conducting usability testing that involves participants who are representative of the audience for which the website is intended. The HLE2 scores suggest that most states should conduct such testing for the CWF content on their websites.

Two states, Indiana and Minnesota, placed the CWF content on the environmental health page, not the oral health pages, making it less likely that a consumer audience would find the content. When users encounter websites that are difficult to navigate or if users cannot find the information they are seeking, they may abandon their search. If information about CWF is missing or not readily visible, users are left without a credible source of information about the best and most cost-effective way to prevent dental caries. This is especially troubling because it also increases the chances users may search elsewhere and find information that is not evidence-based [43].

As SHDs review their CWF web content, the increasing popularity of bottled water also should be a consideration. Many Americans regularly consume bottled water. In a 2019 survey of U.S. adults, 25% said they always chose bottled water when they consumed water with meals at home [52]. This percentage was even higher among non-white Americans, who are at higher risk of dental caries [52]. Consumers may not be aware that many brands of bottled water do not contain sufficient fluoride to offer a decay-prevention benefit [53]. In a study of water-drinking habits among Latino residents of southern Arizona, researchers expressed concern that “fluoride-free bottled water consumption is common in this region, raising concern that families are not receiving adequate fluoride to promote dental health [54].” Although the U.S. Food and Drug Administration permits bottled water companies that add fluoride to cite this as a potential benefit on their label [55], the agency does not require bottled water companies to disclose the amount of naturally occurring fluoride in their products [56]. For this reason, SHD’s should raise awareness that bottled water does not necessarily provide sufficient fluoride. Although our methods did not include a broad review of bottled water-related content on SHDs’ websites, it was encouraging to discover a Minnesota Department of Health web page that educated visitors about bottled water and its fluoride content [57].

Given the prevalence of dental caries, especially among those with lower income and education, SHD’s should do more to increase the public’s understanding of fluoride’s preventive benefits. In this time of growing misinformation about fluoridation, SHDs should be proactive by posting CWF information online that is easily accessible and understandable. States can review our findings and revise their SDH websites to address shortcomings.

## Conclusions

Despite decades of evidence supporting CWF’s role in caries prevention, many Americans are unaware of the benefits of drinking fluoridated tap water. The public deserves CWF information that is accessible and written in plain language so they can understand the benefits. Because SHDs are funded by taxpayers, these departments have an obligation to serve as a key resource for communicating this health-promoting information. These agencies should explore ways to improve their website’s CWF content.

## Supporting information

S1 File (XLSX)

S2 File (PDF)
