# Supplementary material for: Seeking community water fluoridation information on state health department websites
Source: PLoS One. 2021 May 20;16(5):e0251139. doi: 10.1371/journal.pone.0251139 (PMC8136706; doi:10.1371/journal.pone.0251139)
Supplement: S2 File — (PDF) [file pone.0251139.s002.pdf]

(**Note:** Wakefield logo is republished from [52] under a CC BY license, with permission from Wakefield Research, original copyright 2008.)

American Fluoridation Society Questionnaire
QuickRead Report
February 2019

METHODOLOGY

An online survey of 1,000 US adults, ages 18+

CONTENT QUESTIONS

1. When you eat meals at home, how often, if ever, do you drink water while you eat?

Response	
All the time	42%*
Some of the time	39%
Rarely	14%
Never	5%
**QuickFacts±**	
Frequently	81%
Rarely or Never	19%

2. When you drink water with your meals at home, which of the following best describes the type of water you drink?

Response	
I always drink tap water	19%
I usually drink tap water	20%
I drink tap and bottled water evenly	18%
I usually drink bottled water	18%
I always drink bottled water	25%
**QuickFacts±**	
Drinking primarily tap	39%
Drinking primarily bottled	43%

* All decimals are rounded to the nearest percentage point. This may result in certain numerical totals adding up to slightly more or slightly less than 100%.
± Data under "QuickFacts" were derived from the responses, not included as response options that were read during fielding. We include QuickFacts in instances where we feel they will be helpful.

Additional observations about the responses to these questions:

- **Q-1:** Non-parents were much more likely (23%) than were parents (12%) to say they drink water rarely or never with meals served at home.
- **Q-2:** Non-white Americans were more likely (30%) to say they always drink bottled water than were white Americans (24%).

Methodological Notes:

The American Fluoridation Society Survey was conducted by Wakefield Research (www.wakefieldresearch.com) among 1,000 nationally representative U.S. adults ages 18 and older, between January 28th and February 4th, 2019, using an email invitation and an online survey. Quotas have been set to ensure reliable and accurate representation of the U.S. adult population ages 18 and older.

Results of any sample are subject to sampling variation. The magnitude of the variation is measurable and is affected by the number of interviews and the level of the percentages expressing the results. For the interviews conducted in this particular study, the chances are 95 in 100 that a survey result does not vary, plus or minus, by more than 3.1 percentage points from the result that would be obtained if interviews had been conducted with all persons in the universe represented by the sample.
